# Lethal and Sublethal Effects of Chlorpyrifos on Biological Traits and Feeding of the Aphidophagous Predator *Harmonia axyridis*

**DOI:** 10.3390/insects11080491

**Published:** 2020-08-01

**Authors:** Muhammad Asim Rasheed, Muhammad Musa Khan, Muhammad Hafeez, Jing Zhao, Yasir Islam, Shahzaib Ali, Shakeel Ur-Rehman, Um e-Hani, Xingmiao Zhou

**Affiliations:** 1 Hubei Insect Resources Utilization and Sustainable Pest Management Key Laboratory, College of Plant Science and Technology, Huazhong Agricultural University, Wuhan 430070, China; asimrasheed@webmail.hzau.edu.cn (M.A.R.); zhao-jing@mail.hzau.edu.cn (J.Z.); yasirislam2143@gmail.com (Y.I.); shahzaibali@webmail.hzau.edu.cn (S.A.); shakeel.entomologist@hotmail.com (S.U.-R.); 2 Key Laboratory of Bio-Pesticide Innovation and Application Guangdong Province, South China Agricultural University, Guangzhou 510642, China; drmusakhan@outlook.com; 3 Engineering Research Center of Biocontrol, Ministry of Education Guangdong Province, Guangzhou 510640, China; 4 State Key Laboratory Breeding Base for Zhejiang Sustainable Pest and Disease Control, Institute of Plant Protection and Microbiology, Zhejiang Academy of Agricultural Sciences, Hangzhou 310021, China; hafeez_203@yahoo.com; 5 Laboratory of Biological Control of Sustainable Pests, Department of Entomology, College of Plant Protection, China Agriculture University, Beijing 100083, China; um-e-hani09@hotmail.com

**Keywords:** *Harmonia axyridis*, chlorpyrifos, lethal and sublethal effects, toxicity, life table parameters, two-sex life table

## Abstract

Except of pest control, insecticides have shown adverse effects on natural enemies as well. Thus, risk assessment of pesticides for biological control agents is critical for effective use in integrated pest management (IPM) schemes. In the present study, the lethal and sublethal effects of chlorpyrifos, a commonly used insecticide that may negatively affect biological control agents, were evaluated on a non-target predator, the Asian ladybeetle *Harmonia axyridis*. Previous studies have reported on lethal concentrations, but the effects of sublethal concentrations remain unclear. Lethal and sublethal concentrations of chlorpyrifos were applied to third instar larvae of *H. axyridis*, and different growth and developmental parameters were measured. Treatment with LC_10_ (4.62 mg a.i. L^−1^) significantly shortened the developmental period of third instar larvae, whereas it significantly prolonged those of fourth instar larvae and pupa. Treatment with LC_30_ (9.59 mg a.i. L^−1^) significantly increased the larval and pupal developmental period compared with that of the control, whereas feeding potential, female fecundity, and adult longevity significantly decreased after LC_10_ and LC_30_ treatment. The pre-oviposition period significantly increased compared with that of the control. Population growth parameters, the finite (*λ*) and intrinsic rate of increase (*r*) and the net reproductive rate (*R*_0_), decreased following exposure to sublethal concentrations of chlorpyrifos. According to the results, the use of chlorpyrifos in IPM schemes requires further research because even sublethal concentrations of this insecticide were harmful to *H. axyridis* population growth.

## 1. Introduction

Predators play a vital role in the regulation of a wide range of pests in agro-ecosystems [1,2] as they can control different insect pests from different families [3]. Many ladybeetle species (Coleoptera: Coccinellidae) are gregarious predators of several pest species, such as aphids [4,5], whiteflies [6], mealybugs [7], mites [8], and scale insects [9]. Coccinellid predators are particularly useful as biological control agents because both adults and larvae are predaceous. Owing to its global distribution and extensive ability to interrupt agricultural ecosystems, the Asian ladybeetle *Harmonia axyridis* (also known as the harlequin ladybird) is an important predator used in insect-based integrated pest management (IPM) schemes [10,11,12]. Owing to the highly polyphagous nature of *H. axyridis*, this species consumes many species of aphid [13,14]. Feeding potential, voracity, and reproduction rate make this predator an effective biological control agent for different IPM strategies. This beetle is available commercially for insect pest management [15]. Previous studies have shown the effectiveness of *H. axyridis* for the biological control of different pests, such as the apple aphid *Aphis spiraecola* in apple orchards [16], yellow pecan aphid *Monelliopsis pecanis* in pecan orchards [17,18], Asian citrus psyllid *Diaphorina citri* [19], European corn borer *Ostrinia nubilalis* in sweet corn [20], root weevil *Diaprepes abbreviatus* [21], and brown citrus aphid *Toxoptera citricida* [22]. Therefore, *H. axyridis* is an effective biological control agent when used in IPM schemes [11,23].

In addition to releasing biological control agents, IPM strategies commonly include the use of insecticides [24]. However, extensive application of insecticides may cause environmental pollution and insecticide resistance and can also negatively affect natural enemies [25,26,27]. Globally, several insect pest species have shown resistance to generally used insecticides [27,28,29,30,31] and both toxic and sublethal effects have been observed on non-target predators [32,33,34]. Thus, it is necessary to fully understand the negative effects of an insecticide on natural enemies when planning pest control measures within an IPM scheme.

Many studies have focused on the deleterious effects of different insecticides on non-specific arthropods [27,35]. However, most of these studies evaluated the lethal (short term) toxicity of insecticides but did not evaluate the indirect consequences of sublethal effects, which may impair, e.g., the pre-adult development, adult longevity, feeding capacity, female fecundity, and pre-oviposition period of biological control agents [27,35,36,37]. The population growth rate is an important statistical parameter that can be used to comprehensively assess pesticide toxicity [38]. Therefore, life table analysis is an important technique for evaluating the population growth rate and sublethal effects of an insecticide on non-target natural enemies [39].

Chlorpyrifos is a broad-spectrum, non-systemic, synthetic organophosphate that acts as a cholinesterase inhibitor through different exposure routes. In 1969, commercial manufacturing of this insecticide began, since then chlorpyrifos has been used for different purposes. Commonly, this insecticide is used on farms to protect fruit trees, cotton, and corn against insect pests [40]. Similar to other phosphorus compounds, chlorpyrifos induces oxidative stress, damages DNA, and inhibits acetylcholinesterase (AChE) [41]. Adverse effects from the use of this insecticide include negatively affecting non-target beneficial arthropods, such as coccinellid predators e.g., the convergent ladybeetle *Hippodamia convergens* [42], pollinators [43], parasitoid wasps e.g., *Trichogramma brassicae* [44] and other natural enemies e.g., the dipteran parasitoids *Eibesfeldtphora trilobata* and *Apocephalus setitarsus* [45].

The sublethal effects of exposure to chlorpyrifos at the population level of *H. axyridis* have not been previously evaluated. The present study was designed to evaluate the toxicity and sublethal effects of chlorpyrifos on life table parameters of the non-target predator *H. axyridis*, including pre-adult development, feeding potential, survival rate, female fecundity, and male and female adult longevity. For this purpose, an age-stage, two-sex life table technique was applied to provide a more robust understanding of the effects of using chlorpyrifos for pest control in IPM schemes.

## 2. Materials and Methods

### 2.1. Insect Culture

A population of *H. axyridis* was reared on the soya bean aphid *Aphis glycines* (Hemiptera: Aphididae). The mass cultures of *A. glycines* and *H. axyridis* were maintained at 23 ± 2 °C and 68 ± 5% RH, under a 16:8 light:dark photoperiod. *A. glycines* colonies were established on faba bean (*Vicia faba* L.) plants. Faba bean plants were grown in mesh-covered cages (34 cm height × 60 cm length × 44 cm width). *H. axyridis* adults were also reared in mesh-covered cages where the females laid eggs on the leaves of bean plants, and these eggs hatched to larvae and then grew to adults. To establish new colonies of *A. glycines*, fresh faba bean plants were kept in cages with an old plant that was highly infested with *A. glycines*.

Adults of *H. axyridis* were kept separately in pairs in Petri dishes. The sex of adults was determined based on the morphology of the last abdominal segment [46]. Adults were provided with sufficient aphids for successful egg laying. Paired adults were transferred to new Petri dishes (10 mm depth × 100 mm diameter) when egg patches were found. Each neonate was maintained in a single Petri dish to avoid cannibalism and provided with sufficient aphids until they developed to the desired stage for the experiment.

### 2.2. Acute Toxicity Determination

Toxicity bioassays were carried out through the topical application of chlorpyrifos (technical grade chlorpyrifos (95%) was purchased from Dow Agro Sciences, Shanghai, China). Acute toxicity was evaluated on third instar larvae (<24 h) by treating them with seven concentrations of chlorpyrifos (3.12, 6.25, 12.50, 25, 50, 100, and 200 mg L^−1^) prepared by diluting chlorpyrifos in acetone to determine the sublethal concentrations. For every concentration, four replicates were established each containing 15 individuals. Larvae were placed in a small glass tube and immobilized with a small amount of CO_2_ for 4–5 s, and the ventral side of the abdomen of each larva was topically treated with 1 µL of chlorpyrifos solution with a micro-applicator (Burkard, Rickmansworth, UK). Larvae in the control group were treated with 1 µL acetone. Individuals from treatment and control groups were reared in a controlled condition chamber at 23 ± 2 °C, 68 ± 5% RH, under a 16:8 h light:dark photoperiod, and live aphids were provided ad libitum. After treatment, larvae were checked daily to record mortality until either they died or developed to the next stage, and after 72 h of treatment, acute mortality data were recorded. Larvae showing no movement when softly pushed with a soft brush were considered to be dead [47].

### 2.3. Evaluation of Sublethal Effects on Life Table of H. axyridis

To determine life table parameters, approximately 600 eggs of *H. axyridis* (<24 h) were kept in Petri dishes (10 mm depth × 100 mm diameter) following a technique defined previously [48,49]. Two treatment groups (LC_10_: 4.62 mg a.i. L^−1^ and LC_30_: 9.59 mg a.i. L^−1^) and one control group (treated with acetone) were established. In each group, 150 newly developed third instar larvae were selected, and each larva was considered as a single replicate [50]. The LC_10_ and LC_30_ values were calculated from acute toxicity bioassays of chlorpyrifos. Treated larvae were supplied with live aphids on fresh leaves, and mortality data were recorded daily until the larvae developed to the next instar. After adult emergence in all treatments, males and females were kept in pairs to record mortality, pre-oviposition period, female fecundity, and adult longevity data.

### 2.4. Assessment of Feeding Potential of H. axyridis

To assess the feeding ability of *H. axyridis* larvae and adults following insecticide exposure, approximately 30 third instar larvae were treated with acetone as a control group, 50 third instar larvae were treated with the LC_10_ concentration (4.62 mg a.i. L^−1^), and 50 third instar larvae were treated with the LC_30_ concentration (9.59 mg a.i. L^−1^), considering each larva as one replicate. Treated larvae were maintained in Petri dishes and sufficient adult aphids were provided ad libitum. Dead larvae were removed, and 50 adult aphids were provided daily to each replication to feed the treated larvae. The number of aphids consumed by larvae per day was recorded daily until the larvae developed to the next instar or to the adult stage. For *H. axyridis* adults, 70 adult aphids were provided to each adult lady beetle daily, and per day aphid consumption of adults (developed from the larvae treated previously) was recorded separately for males and females.

### 2.5. Effects of Sublethal Chlorpyrifos Concentrations on Population Growth Parameters

Population growth parameters were also evaluated, including *r*, intrinsic rate of increase (*r* = ln(*R*_0_)/*T*), which denotes the maximum population increase rate. *λ*, finite rate of increase (*λ* = exp (*r*)), which is an expression of the factors responsible for population growth. *R*_0_, net reproduction rate (*R*_0_ = *Σl_x_m_x_*), is the value of the population growth rate, including the female offspring produced by a female in one generation, and *T*, mean generation time (*T* = *Σl_x_m_x_*/*R*_0_), is the average time interval between the birth of two consecutive generations.

### 2.6. Effect of Chlorpyrifos Sublethal Concentrations on Demographic Parameters

In addition to the differences between the developmental periods of life stages, demographic parameters were also recorded, including *s_xj_* (age-stage-specific survival rate), where *x* denotes age and *j* denotes stage; *l_x_* (age-specific survival rate), a simple form of *s_xj_*, which is an estimation of the age of a newly hatched egg [51]; *f_xj_* (age-stage-specific fecundity), which is the female fecundity for a given number of days at age *x* and stage *j*; *m_x_* (age-specific fecundity), which is the number of eggs laid per individual at age *x, l_x_m_x_* (age-specific maternity), which is the combination of *l_x_* and *m_x_*; *V_xj_* (age-stage-specific reproduction), which is the degree of involvement of each individual in the next generation and *e_xj_* (life expectancy), which is an estimation of the expected survival time of each individual.

### 2.7. Statistical Analysis

PoloPlus [52] was used to calculate concentrations of chlorpyrifos that were lethal and sublethal for third instar larvae in the acute toxicity bioassay. The age-stage two-sex life table model was used to analyze the development of different stages, survival rate, fecundity parameters, pre-oviposition period, and adult longevity [53,54], and the TWOSEX-MS Chart software was obtained from http://140.120.197.173.193 [55]. Standard errors (SEs) and means were calculated based on 100,000 bootstrap iterations [51,56]. The paired bootstrap test was used for comparing all treatments; bootstrap and paired bootstrap tests were analyzed in TWO SEX-MS Chart [55]. Statistics 8.1 was used for calculating the means and SEs for feeding potential data. SigmaPlot 12.0 was used to draw graphs of feeding potential data and the curves of all parameters, including fecundity, survival rate, life expectancy, and reproductive values. And according to completely randomized design, the data of the feeding potential of *H. axyridis* were statistically analyzed using one-way ANOVA (analysis of variance) and their mean values were compared using least significant difference (LSD) tests at the *p* = 0.05 level of significance.

## 3. Results

### 3.1. Chlorpyrifos Toxicity on Third Instar H. axyridis Larvae

Acute toxicity bioassays were carried out to determine the lethal (toxic) and sublethal concentrations of chlorpyrifos for third instar *H. axyridis* (Table 1). After 72 h of treatment, lethal and sublethal concentrations of chlorpyrifos were calculated. The LC_10_, LC_30_, LC_50_, and LC_90_ values for third instar larvae were calculated as 4.62 (mg a.i. L^−1^), 9.59 (mg a.i. L^−1^), 15.90 (mg a.i. L^−1^), and 54.63 (mg a.i. L^−1^), respectively but only sublethal concentrations (LC_10_ and LC_30_) were used in life-table experiments. Control group mortality was observed to be <10%.

### 3.2. Sublethal Effects of Chlorpyrifos on H. axyridis

#### 3.2.1. Effects on Pre-Adult Development

We evaluated the sublethal effects of chlorpyrifos on the developmental period of pre-adult stages (Table 2), male and female adult longevity, pre-oviposition period, and fecundity of *H. axyridis* (Table 3). Results show that the LC_10_ treatment significantly shortened the developmental period of the third instar, whereas the developmental periods of fourth instar larvae and pupa were significantly prolonged compared with those in the control. Larval and pupal development times significantly increased after the LC_30_ treatment.

#### 3.2.2. Effects on Life Table Parameters of *H. axyridis* Adults

Female fecundity and male and female adult longevity were negatively affected by chlorpyrifos exposure. Treatment with LC_10_ and LC_30_ concentrations significantly decreased the female and male adult longevity and female fecundity compared with those of the control. Conversely, the adult pre-oviposition period (APOP) and total pre-oviposition period (TPOP, calculated from eggs hatched) were significantly prolonged after treatment with LC_10_ and LC_30_ (Table 3).

### 3.3. Effects on H. axyridis Population Growth Parameters

The effect of chlorpyrifos on population growth parameters is shown in Table 4. Results show that *r*, *λ*, and *R*_0_ were negatively affected by chlorpyrifos exposure. LC_10_ and LC_30_ treatments significantly reduced the values of *r*, *λ*, and *R*_0_. There was no significant effect on mean generation time.

### 3.4. Effects on H. axyridis Feeding Potential

**Figure 1:**
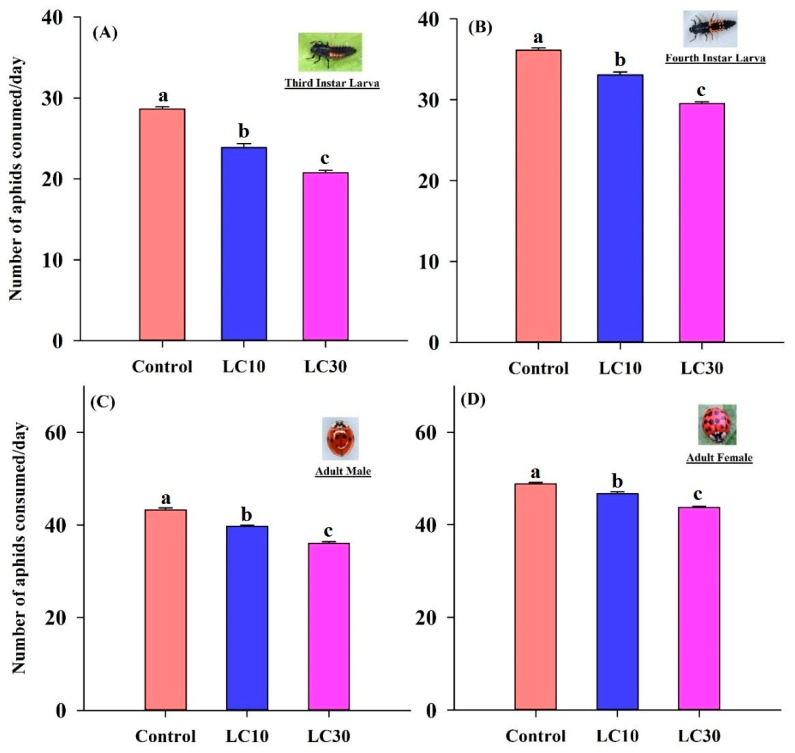
Feeding potential of the pre-adult and adult stages of *H. axyridis* in the control and treatment (LC_10_, LC_30_) groups. (**A**) = feeding potential of 3rd instar, (**B**) = feeding potential of 4th instar, (**C**) = feeding potential of adult male, (**D**) = feeding potential of adult female. Different letters above each bar indicate significant differences between treatments using one-way ANOVA, LSD test (*p* = 0.05 and *n* = 30 (Control), *n* = 50 (LC_10_ & LC_30_)).

Sublethal concentrations of chlorpyrifos negatively affected the feeding potential of third and fourth instar larvae and adults of *H. axyridis* (Figure 1). The results demonstrate that LC_10_ and LC_30_ treatments significantly reduced the feeding potential of third instar (*F*_2,27_ = 44.7, *p* < 0.001) and fourth instar (*F*_2,27_ = 26.9, *p* < 0.001) larvae, adult males (*F*_2,27_ = 109, *p* < 0.001), and adult females (*F*_2,27_ = 8.25, *p* = 0.0016) as compared to that of the control.

### 3.5. Effects on H. axyridis Demographic Parameters

#### 3.5.1. Effect on Survival Rate

**Figure 2:**
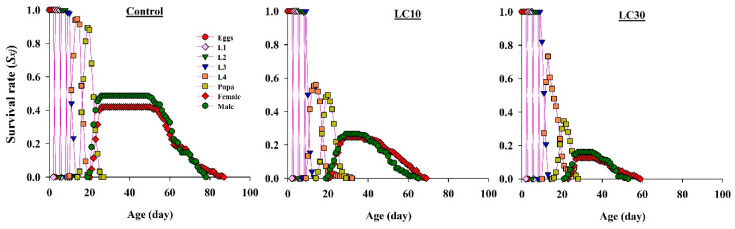
Graphs show *sxj* (age-stage-specific survival rate) values of offspring produced by *Harmonia axyridis* females treated with sublethal chlorpyrifos concentrations.

The *s_xj_* value decreased in the treated populations (Figure 2). Results show that the survival rate of third instar larvae was higher than that in subsequent stages. The highest peaks of *s_xj_* for fourth instar larvae were at 0.94 in the control, 0.56 in LC_10_, and 0.73 in LC_30_. The pupal stage was more susceptible to increasing insecticide concentration (control: 0.89, LC_10_: 0.49, LC_30_: 0.34). *s_xj_* peaked in the control group (males: 0.48, females: 0.42), whereas this value decreased in treatments with an increase in chemical concentration (LC_10_: 0.26 for males, 0.24 for females; LC_30_: 0.16 for males, 0.12 for females). Mean longevity of females and males in the control was 54 and 56 days, respectively, higher than that in the LC_10_ (43.54 and 45.46 days) and LC_30_ (38.24 and 41.81 days) treatment groups.

**Figure 3:**
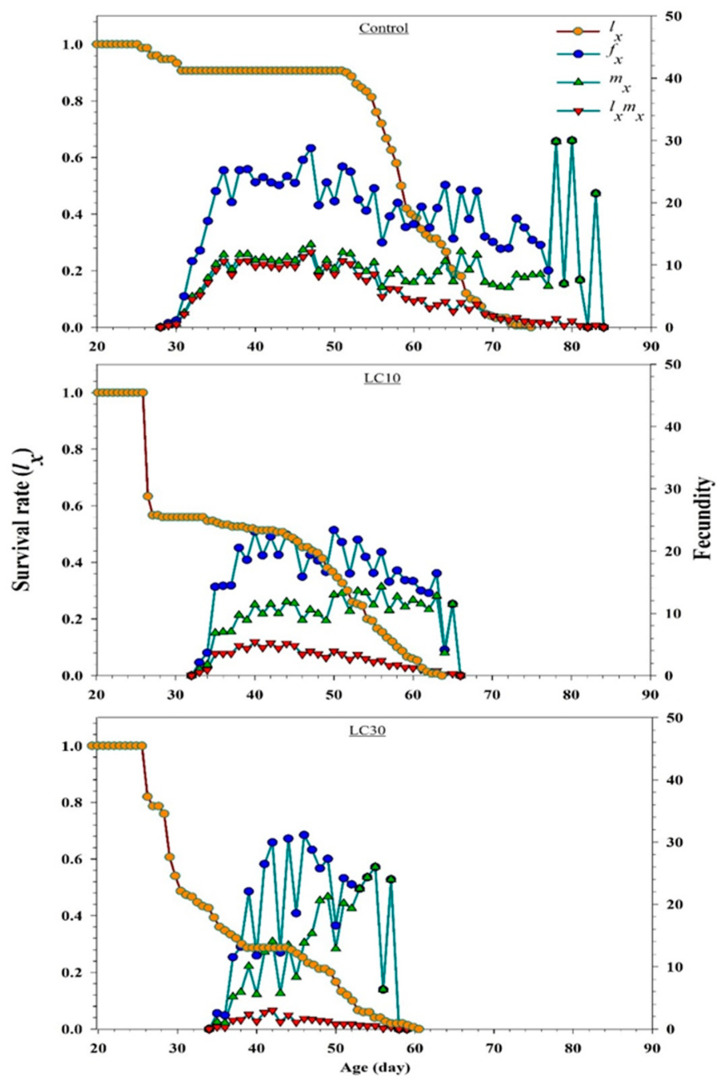
Graphs show *l_x_* (Age-specific survival rate), *f_xj_* (female age-stage-specific fecundity), *m_x_* (the age-specific fecundity of total population) and *l_x_m_x_* (net maternity) values for offspring produced by *H. axyridis* females treated with sublethal chlorpyrifos concentrations.

Graphs of *l_x_* (age-specific survival rate), *f_xj_* (age-stage-specific fecundity), *m_x_* (total fecundity of population), and *l_x_m_x_* (net maternity) are shown in Figure 3. *l_x_* represents a simple form of the *S_xj_* curves. On the 30th day, the slope of the *l_x_* curve of the control group (0.90) was greater than that of the LC_10_ (0.52) and LC_30_ (0.28) curves, which decreased following insecticide treatment. The *f_xj_* (age-stage-specific fecundity, mean number of individuals produced by a female at age x) values were also negatively affected by pesticide treatment. In the control, the highest observed value of *f_xj_* was 29.86 eggs female^−1^ day^−1^ on the 78th day, whereas in the LC_10_ treatment, the *f_xj_* value was 23.36 eggs female^−1^ day^−1^ on the 50th day. The highest calculated *m_x_* value for the control (30 eggs individual^−1^ day^−1^ on the 80th day) was found to be greater than that in LC_10_ (14.28 eggs per individual^−1^ day^−1^ on the 56th day) and LC_30_ (26 eggs individual^−1^ day^−1^ on the 55th day). The *l_x_m_x_* curves depended on *l_x_* and *m_x_* values. The highest peak of *l_x_m_x_* was observed in the control at the age of 47 (12.08), LC_10_ at the age of 46 (6.90), and LC_30_ at the age of 42 (2.99) days.

#### 3.5.2. Effect on Life Expectancy

**Figure 4:**
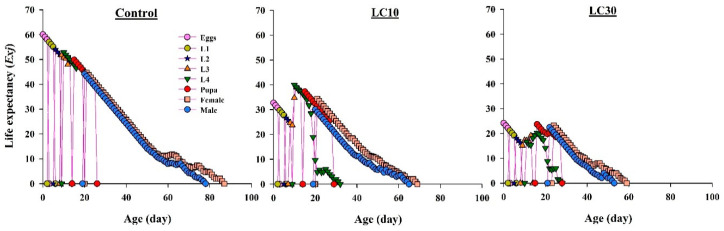
Graphs show *e_xj_* (Life expectancy) values for offspring produced by *H. axyridis* females treated with sublethal chlorpyrifos concentrations.

The graphs in Figure 4 show lower *e_xj_* values for newly hatched eggs in LC_10_ and LC_30_ compared with the value of those in the control. In the control, *e_xj_* was 60 days, which considerably decreased in LC_10_ (32.67 days) and LC_30_ (24.16 days). The *e_xj_* curves showed that the life expectancy of 24-day-old females and males further reduced in the chlorpyrifos-treated population compared with those in the control. The *e_xj_* values of 24-day-old females and males were 41.69 and 40.19 in the control, 31.4 and 25.9 in LC_10_, and in LC_30_ it was further reduced to 23.26 and 20.45.

#### 3.5.3. Effect on Reproduction

**Figure 5:**
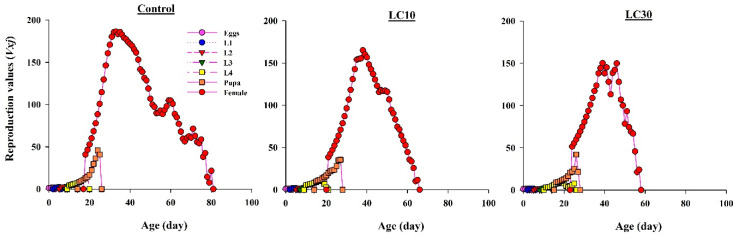
Shows *v_xj_* (Age-stage specific reproduction) values for offspring produced by *H. axyridis* females treated with sublethal chlorpyrifos concentrations.

*v_xj_* (age-stage-specific reproduction) reflects the degree of involvement of each individual in the next generation. The curves show that *v_xj_* values were lower in the chlorpyrifos-treated groups (both LC_10_ and LC_30_) compared with those in the control (Figure 5). The highest observed peak value of *v_xj_* was 186.67 eggs/day (on the 36th day) in the control, 165 eggs/day (on the 38th day) in the LC_10_ treatment, and 150 eggs/day (on the 39th day) in the LC_30_ treatment.

## 4. Discussion

Non-target organisms are directly or indirectly effected by a number of insecticides extensively applied in agriculture for pest control [34,57]. We evaluated the sublethal effects of chlorpyrifos on life table parameters of *H. axyridis*. The LC_50_ value of chlorpyrifos was 15.90 (mg a.i. L^−1^) for the third instar larvae, which is less than the recommended field concentration [42]. The developmental period of third instar larvae was shortened following LC_10_ treatment, whereas LC_30_ treatment prolonged this period. Sublethal concentrations (LC_10_: 4.62 mg a.i. L^−1^, LC_30_: 9.59 mg a.i. L^−1^) of chlorpyrifos prolonged the developmental periods of fourth instar larvae and pupae and significantly negatively affected feeding potential, male and female adult longevity, female fecundity, population growth, and demographic parameters. Similar results were reported when the effect of chlorpyrifos on the generalist predator *H. convergens* was evaluated [42]. Among the different life stages of *H. axyridis*, a high level of toxicity was calculated when third instar larvae were exposed to chlorpyrifos, which shows that this stage was more vulnerable to chlorpyrifos than the adult stage in the present study. Different toxicity levels between life stages have been reported for *H. axyridis* [25,35,58], *Ceraeochrysa cubana* (Neuroptera: Chrysopidae) [59], *Chrysoperla carnea* (Neuroptera: Chrysopidae) [60], and *Adalia bipunctata* (Coleoptera: Coccinellidae). The lower adult susceptibility is due to a high amount of cuticle sclerotization in the integument of adult insects, which reduces insecticide penetration [61].

Age-stage, two-sex life table theory is a comprehensive tool for evaluating the total effects of an insecticide on insect pest populations based on life stage developmental periods, survival, adult longevity, and female fecundity [62]. The results of larval and pupal development show that even lower concentrations of chlorpyrifos are toxic for pre-adult development. Chlorpyrifos treatment prolonged the developmental period of larval and pupal stages in comparison with that of the control because, after treatment, most of the energy in a treated individual was used for detoxification of the applied chemical [63]. In another study, chlorpyrifos treatment also increased the larval and pupal developmental period of *H. convergens* [42]. Similar results were reported in a study showing the adverse effects of chlorantraniliprole on *H. axyridis* [35].

Despite an increase in the pre-oviposition period (adult and total), a strong decrease was observed in adult life span (male and female longevity), feeding potential, and female fecundity. Decreases in the fecundity of three natural enemies, *Orius insidiosus* (Hemiptera: Anthocoridae), *Cycloneda sanguinea* (Coleoptera: Coccinellidae), and *Chauliognathus flavipes* (Coleoptera: Cantharidae) were previously confirmed when chlorpyrifos was evaluated for safety [64]. The reduced fecundity in our results was consistent with that reported in a previous study [42], which revealed a strong decrease in female fecundity of *H. convergens* when exposed to chlorpyrifos. Reduced fecundity was also reported when the natural enemy *Habrobracon hebetor* (Hymenoptera: Braconidae) was exposed to sublethal concentrations of chlorpyrifos [65]. The above mentioned results are all consistent with studies of neonicotinoid insecticides, including clothianidin [66] and acetamiprid [67], reducing the fecundity of coccinellid predators. Decreased fecundity may be the result of ovary deformation caused by pesticide application [68].

Predation ability is also adversely affected after insecticide exposure [69]. Our results were consistent with this, showing that the feeding potential of larvae and adults of *H. axyridis* was significantly reduced when third instar larvae were treated with chlorpyrifos. Similar results were previously reported regarding the decreased foraging time and feeding capacity of other predators, *Macrolophus pygmaeus* (Hemiptera: Miridae), *Coleomegilla maculata*, *Serangium japonicum* and *Hippodamia convergens* (Coleoptera: Coccinellidae) because of neonicotinoid insecticide exposure [69,70,71]. Adult male and female longevity was shortened in *H. convergens* [42] and *H. hebetor* [65] after exposure to chlorpyrifos. These results are similar to those of our study regarding chlorpyrifos exposure significantly reducing male and female longevity. According to these results, *H. axyridis* is very susceptible to chlorpyrifos, specifically during the pre-adult stages. The absolute change in all parameters after chlorpyrifos treatment may have been because of the higher toxicity of chlorpyrifos than that of other insecticides [64].

The comprehensive study of life table parameters is necessary for the assessment of the sublethal effects of an insecticide on insects as well as on the population growth rate [39,72]. Our results demonstrate that population growth parameters *r*, *R*_0_, and *λ* were significantly lower after LC_10_ and LC_30_ treatments, indicating that sublethal concentrations of chlorpyrifos cause damaging effects on the physiology of insects, which are often not observed in the short term [73]. Corroborating these results, a decrease in *r*, *λ*, and *R*_0_ values was observed in chlorpyrifos-exposed *H. convergens* populations [42]. Similar results were reported when the toxic effects of chlorpyrifos were observed on the population growth and biological activity of *Bracon hebetor* (Hymenoptera: Braconidae) [74].

Our results confirm the negative effects of chlorpyrifos on demographic parameters of *H. axyridis* by showing the deleterious effects on population growth rate. Specifically, *s_xj_* was significantly lower after chlorpyrifos treatment, whereas *f_xj_* and *m_x_* values were also decreased in LC_10_ and LC_30_ treatments. A similar tendency was observed in the reproduction value, *v_xj._* In addition, a strong decrease was found in population growth, *e_xj_*. Sublethal effects were also observed in later stages; *e_xj_* values of freshly hatched eggs were higher for the control than the treatment groups due to high chlorpyrifos stress. Our results show that chlorpyrifos application exerted negative effects on the feeding, survival rate, reproduction, and development of *H. axyridis*. Further work is required to evaluate the genetic changes that occur following exposure to sublethal concentrations of chlorpyrifos.

Except of being an effective biological control agent, *H. axyridis* is also known as an invasive species in some countries of the world [10]. It is native to central and eastern Asia occurring in Korea, Japan, Mongolia, China, Russian Far East [75]. In some regions, it has become invasive, producing negative socio-economic and ecological effects [76]. It was introduced to North America for biological control of coccids and aphids [77] but in 1988, its dispersal was observed in the wild areas [78]. It was introduced across Europe as a biological control agent of aphids [75]. Because of its excellent dispersal abilities, it invaded to Britain also [79]. It is a successful invader due to polyphagous nature, excellent capacity to disperse and establish and flexibility of its immune system [80].

## 5. Conclusions

In the present study, chlorpyrifos exhibited high toxicity to *H. axyridis* because it increased the pre-adult developmental period, pre-oviposition period, and lowered male and female adult longevity, female fecundity, and feeding potential. Treatment with this insecticide negatively affected both life table and demographic parameters. According to our results, we suggest that the field application of chlorpyrifos can damage *H. axyridis* populations either in the short or long term, so the use of this insecticide needs more attention and care in IPM schemes.

## Figures and Tables

**Table 1:** Acute toxicity of chlorpyrifos on 3rd instar larvae of *Harmonia axyridis*.

Insecticide	Concentration (95% CL)^−1^ mg (a.i.) L^−1^						
	N	LC_10_	LC_30_	LC_50_	LC_90_	Slope ± SE	*χ*^2^ (Df)
Chlorpyrifos	360	4.62	9.59	15.90	54.63	2.391 ± 0.225	5.822 (4)
		(2.31–6.92)	(6.27–12.89)	(11.70–21.19)	(37.66–102.01)		

N = number of third instar larvae of *H. axyridis* treated with chlorpyrifos.

**Table 2:** Sublethal effects of chlorpyrifos on the developmental period (mean ± SE) of pre-adult stages of *H. axyridis*.

Treatments	Development Period of Immature Stages		
	Third Instar Larva (Day)	Fourth Instar Larva (Day)	Pupa (Day)
Control	2.68 ± 0.07 b	5.31 ± 0.06 c	5.74 ± 0.06 c
LC_10_	2.13 ± 0.10 c	6.46 ± 0.12 b	6.70 ± 0.13 a
LC_30_	2.95 ± 0.08 a	7.44 ± 0.22 a	6.14 ± 0.19 b

Means followed by the same letters in the same column are not significantly different based on the paired bootstrap test at the 5% significance level. 150 insects were used for each treatment.

**Table 3:** Sublethal effects of chlorpyrifos on the life parameters (mean ± SE) of *H. axyridis* adults treated with insecticide from the third instar larval stage.

Treatments	Female Adult Longevity (d)	Male Adult Longevity (d)	APOP (d)	TPOP (d)	Fecundity (Eggs/Female)
Control	65.43 ± 1.16 a	63.89 ± 0.9 a	9.76 ± 0.12 c	33.14 ± 0.21 c	694.84 ± 17.28 a
LC_10_	55.41 ± 1.36 b	50.02 ± 1.13 b	11.76 ± 0.14 b	36.27 ± 0.32 b	379.03 ± 24.21 b
LC_30_	47.26 ± 1.55 c	44.46 ± 0.92 c	12.61 ± 0.30 a	38.50 ± 0.46 a	229.06 ± 36.88 c

Means followed by the same letters in the same column are not significantly different based on the paired bootstrap test at the 5% significance level. 150 insects were used for each treatment.

**Table 4:** Sublethal effects of chlorpyrifos on the population growth parameters (mean ± SE) of *H. axyridis* adults exposed to insecticide from the third instar larval stage.

Treatments	Population Growth Parameters			
	(*r*)	(*λ*)	(*R*_0_)	(*T*)
Control	0.12 ± 0.002 a	1.13 ± 0.002 a	290.33 ± 28.60 a	43.92 ± 0.33 a
LC_10_	0.10 ± 0.003 b	1.10 ± 0.004 b	93.36 ± 14.53 b	44.40 ± 0.50 a
LC_30_	0.07 ± 0.006 c	1.07 ± 0.006 c	27.48 ± 7.43 c	44.22 ± 0.75 a

Means followed by the same letters in the same column are not significantly different as calculated using the paired bootstrap test at the 5% significance level. *r* = Intrinsic rate of increase day^−1^; *λ* = Finite rate of increase day^−1^; *R*_0_ = Net reproductive rate (offspring per individual); *T* = Mean generation time.
